# Evaluation of the effects of the Arm Light Exoskeleton on movement execution and muscle activities: a pilot study on healthy subjects

**DOI:** 10.1186/s12984-016-0117-x

**Published:** 2016-01-23

**Authors:** Elvira Pirondini, Martina Coscia, Simone Marcheschi, Gianluca Roas, Fabio Salsedo, Antonio Frisoli, Massimo Bergamasco, Silvestro Micera

**Affiliations:** Bertarelli Foundation Chair in Translational Neuroengineering, Center for Neuroprosthetics and Institute of Bioengineering, École Polytechnique Fédérale de Lausanne (EPFL), Lausanne, Switzerland; Wyss Center for Bio-and Neuro- Engineering, Geneva, Switzerland; PERceptual Robotics Laboratory, Scuola Superiore Sant’Anna, Pisa, Italy; The Biorobotics Institute, Scuola Superiore Sant’Anna, Pisa, Italy

**Keywords:** Arm exoskeleton, Robotic therapy, Upper limb neuro-rehabilitation, Reaching movements

## Abstract

**Background:**

Exoskeletons for lower and upper extremities have been introduced in neurorehabilitation because they can guide the patient’s limb following its anatomy, covering many degrees of freedom and most of its natural workspace, and allowing the control of the articular joints. The aims of this study were to evaluate the possible use of a novel exoskeleton, the Arm Light Exoskeleton (ALEx), for robot-aided neurorehabilitation and to investigate the effects of some rehabilitative strategies adopted in robot-assisted training.

**Methods:**

We studied movement execution and muscle activities of 16 upper limb muscles in six healthy subjects, focusing on end-effector and joint kinematics, muscle synergies, and spinal maps. The subjects performed three dimensional point-to-point reaching movements, without and with the exoskeleton in different assistive modalities and control strategies.

**Results:**

The results showed that ALEx supported the upper limb in all modalities and control strategies: it reduced the muscular activity of the shoulder’s abductors and it increased the activity of the elbow flexors. The different assistive modalities favored kinematics and muscle coordination similar to natural movements, but the muscle activity during the movements assisted by the exoskeleton was reduced with respect to the movements actively performed by the subjects. Moreover, natural trajectories recorded from the movements actively performed by the subjects seemed to promote an activity of muscles and spinal circuitries more similar to the natural one.

**Conclusions:**

The preliminary analysis on healthy subjects supported the use of ALEx for post-stroke upper limb robotic assisted rehabilitation, and it provided clues on the effects of different rehabilitative strategies on movement and muscle coordination.

## Background

In 2010, 8.2 million of people in Europe were affected by a stroke, with a total cost of about 64 billion euro per year [1]. With the increasing of life duration, it is expected that the stroke related disabilities in western societies would be ranked to the fourth most important causes of disability in 2030 [2]. Impairments in reaching movements occur in about two-thirds of stroke survivors: upper limb functions are altered in the 73–88 % of first time stroke survivors, and in the 55–75 % of chronic post-stroke patients [3, 4]. Indeed, in most of the cases post-stroke subjects remain unable to use their paretic limb to execute even basic actions, losing their independence in carrying out the everyday activities.

Rehabilitation has the ultimate outcome to reintroduce the patient as an active participating member in the society [5]. Rehabilitative interventions based on task-oriented repetitive movements have showed to improve muscle strength and movement coordination in patients with neurological impairments [6, 7], pointing out how intensive rehabilitation can have long-term benefits in patients with moderate-to-severe impairment, even years after a stroke [8]. For the above reasons, in the last decades, robotic-based rehabilitation, which allows improving the intensity and the repeatability of the rehabilitative treatment, has become very widespread. Indeed, robots can both provide quantitative measures of motor performances for the assessment of motor improvement [9] and precisely control the execution of complex motor tasks [10], producing measured levels of assistance or precise repeatable force patterns [11], and allowing the design of rehabilitative interventions that continuously challenge the patient’s neuromuscular system [12].

Exoskeletons are wearable robotic devices where the limb is enclosed in an actuated robotic suit conform to the configuration of the limb [13]. They can be designed to cover as many degrees of freedom (DoFs) as the human limbs and to precisely determine the position and the delivered assistance torque at each articular joint [14]. Exoskeletons offer several advantages over end-effector-based therapy robots, in particular for upper limb rehabilitation: they enlarge the task space to three dimensions, they follow the arm in its natural workspace with no restrictions, and they allow the independent or synergistic motion of shoulder, elbow, and wrist joints during the execution of functional movements [15].

Despite they improved the versatility of the robotic rehabilitation, the superiority of exoskeleton aided-rehabilitation over conventional rehabilitative treatment has still to be proven [9, 15–18]. This could be ascribable to a not optimal use of the robotic devices due in particular to a lack of knowledge about the impact of the different rehabilitative strategies on motor and muscle organization [19].

The optimal use of a robotic device can be achieved only after a deep characterization of its functionality. Therefore, in this study we aimed at evaluating the use of a new upper limb exoskeleton, the Arm Light Exoskeleton (ALEx) [20] for robot-aided neurorehabilitation, and at investigating the effects of some common rehabilitative strategies adopted in robot-assisted training. In our previous work [21], ALEx was evaluated by characterizing its influence on end-effector (EE) kinematics and on muscle activity and coordination (studying muscle synergies). Our preliminary results showed that the use of ALEx in the passive modality (i.e. with the compensation of weight, friction and inertia, but without the assistance of the robot during movement execution) does not interfere with movement execution and just slightly modifies muscle activity and coordination.

In order to give a more complete evaluation of the effects of ALEx on the execution of reaching movements and of the strategies adopted by the subjects while using it, in this work we extended our preliminary analysis looking at the effects of the exoskeleton on joint kinematics and on motoneuronal (MN) activity in the spinal circuitries [22–25]. Furthermore, we tested the reliability of the device across different days.

Finally, in order to provide deeper insights on the effects of different rehabilitative strategies, we evaluated the execution of the movements, muscle and spinal activity during passive and active training. The active training (i.e., with the assistance of the robot during movement execution) can be supported by many desired trajectories (such as linear trajectories or pre-recorded trajectories from healthy limbs), but there is no evidence about the effects of the trajectories in promoting muscle activity and motor plasticity [19]. Hence, we tried assessing, in a smaller cohort of subjects, the differences in the induced muscle activity and coordination between the execution of passive linear and natural trajectories during point-to-point movements.

We believe that this study provides a complete characterization of ALEx, and technological support and theoretical insights to enhance the efficacy of robot-aided rehabilitation.

## Methods

### Description of the Arm Light Exoskeleton

**Fig. 1:**
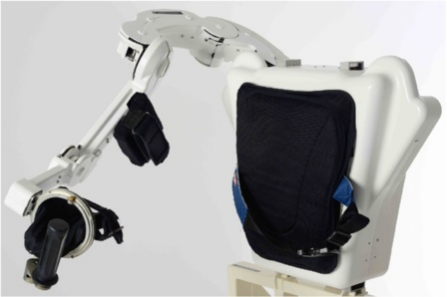
ALEx, the new Arm Light Exoskeleton

ALEx (Fig. 1) is a six DoFs mechanically compliant exoskeleton for the human upper limb: four DoFs are sensorized and actuated (the shoulder abduction, SH-Abd, rotation, SH-Rot, and flexion, SH-Flx, and the elbow flexion, EL-Flx), and two DoFs are sensorized and passive (the forearm prono-supination, FO-Pro, and the wrist flexion, WR-Flx). A peculiarity of the design of ALEx is the patented implementation of the shoulder rotation mechanism [26, 27] that makes use of a remote center of rotation. This solution allows a kinematic isomorphic to that of the human arm and, thus, the alignment of its joints axes with the corresponding axes of the human articular joints*.*

ALEx can reach about 90 % of the natural workspace of the human arm without singularities, covering the following range of motion for each DoF: 0 to 110 deg for SH-Abd, −40 to 60 deg for SH-Rot, 10 to 155 deg for SH-Flx, 0 to 160 deg for EL-Flx, −90 to 90 deg for FO-Pro, and −50 to 50 deg for WR-Flx (for each DoF, the zero is set in the configuration with the upper arm segment parallel to the trunk and the elbow joint flexed at 90 deg). In all postures within its workspace, ALEx allows the generation of a maximum continuous interaction force at the EE of 50 N with a maximum peak force of 100 N, and a maximum continuous joint torque of 40 Nm with a maximum peak torque of 80 Nm and a maximum joint speed of 500 deg/s.

ALEx is equipped with four brushless torque motors located in its backpack, four optical incremental encoders located at the motors’ axes, and patented absolute angular position sensors located at the joints [26, 27]. A compliant cable transmission implements the transmission of the torques from the motors to the joints. As consequence, the exoskeleton’s arm and the entire device weigh only 4.5 and 16.4 Kg, respectively.

The robot can be operated either in force mode, providing desired input forces to the EE or joint torques to each joint, or in compliant position mode, providing desired trajectories with the associated stiffness to the EE or to the joints.

The high-level control includes the possibility to use ALEx in three modalities: *i*) passive*,* in which the subject moves the upper limb and the robot measures the movements, *ii*) assistive, in which the robot guides the upper limb of the subject during movement execution, and *iii*) “assisted-when-needed”, in which the robot guides the arm of the user in the target position if the user does not initiate the movement in less than three seconds. In all modalities, the weight of its moving links (gravity compensation), the friction of its mechanical transmissions (friction compensation), and the inertia of its moving parts (compensation of masses and inertias of links and motors) are compensated by the control.

### Participants and conditions

Six right-handed healthy young subjects (one female, five males, age 26.5 ± 3.4, weight 76.5 ± 9.1 kg, and height 1.77 ± 0.03 m) were enrolled in the study. They did not present any evidence or known history of skeletal or neurological diseases, and they exhibited intact joint range of motion and muscle strength. The study was carried out in the Translational Neural Engineering Laboratory at the École Polytechnique Fédérale de Lausanne, Switzerland (EPFL). It was approved by the EPFL Brain Mind Institute Ethics Committee for Human Behavioral Research, and the recordings were carried out in agreement with the Declaration of Helsinki. At the beginning of each experimental session the participants were informed of the procedure and they signed an informed consent, which included the consent to the use of all data collected during the experiment in scientific publications.

The evaluation was performed in three sessions in order to avoid muscle fatigue and to evaluate the reliability of ALEx. In day 1, the execution of free reaching movements and movements executed wearing the exoskeleton in passive modality were tested. Part of these results have already been reported in [21]. In day 2, the reaching movements were executed wearing the exoskeleton in passive and assistive modality (i.e., compliant position mode at the EE), in order to assess whether muscle activity and coordination were preserved during active training. In day 3, we evaluated in a small cohort of subjects (i.e., only three of the six subjects could participate) the possible assistive controls of the exoskeleton and their effects on muscle activity and coordination. During this last session, the exoskeleton was used in passive modality and then in assistive modality with two different control strategies: the joint control and the EE control, *i.e.* by respectively proposing the joint angular or the EE trajectories previously performed and recorded in the passive modality. Hence, the EE trajectories proposed in the assistive modality of day 2 and day 3 differed: in the first case, the trajectories at the EE were straight lines between the initial position and the target, whereas, in the second case, the trajectories at the EE were the natural movements previously performed by the subject in the passive modality.

Table 1 summarizes the conditions and the metrics computed for each condition.

**Table 1:** Experiment conditions and metrics

Condition description	nMD	Pace	nPK	Angular excursions	RMS_EMG_	Muscle synergies	MN activity	MN activity of muscle synergies
Free movements	X	X	X	X	X	X	X	X
Passive modality day 1	X	X	X	X	X	X	X	X
Passive modality day 2	X	X	X	X	X			
Passive modality day 3	X	X	X	X	X	X	X	X
Assistive modality				X	X	X	X	X
Assistive modality with EE control				X	X	X	X	X
Assistive modality with joint control				X	X	X	X	X

X indicates that the corresponding metric or analysis has been applied to the respective condition

### Experiment setup

**Fig. 2:**
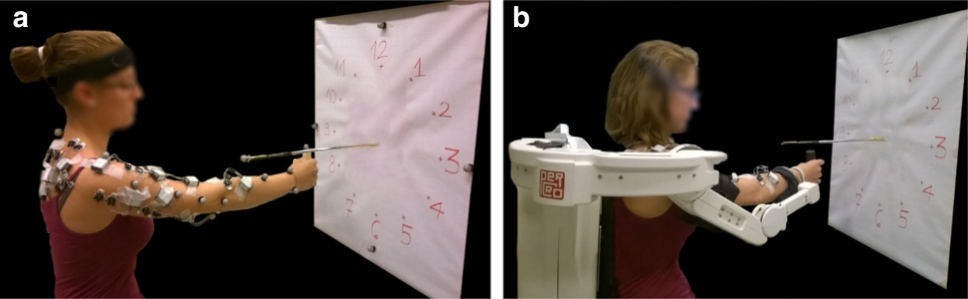
The experimental setup. **a** The experimental setup for free reaching movements. **b** The experimental setup for the conditions with the exoskeleton

In each session and condition, the subject seated behind a target panel frame, with the center of the target panel aligned with the acromion of the right arm (Fig. 2). The distance between the subject and the panel corresponded to the subject’s arm length. The panel frame contained 12 targets arranged in a clock-like fashion, placed 20 cm from its center. The starting position was located mid-way between the center of the target panel and the acromion, at the same height. The subject had to reach from the starting position each of the 12 targets on the target panel frame, and to move back from each target to the starting position with the occurrence of a metronome tone at a frequency of 40 beats per minute (bpm), which corresponds to a movement speed of 0.24 m/s. The metronome rhythm was chosen as a compromise between a low/moderate speed to resemble the reaching speed of elderly and post-stroke subjects (the final users of the device) [28] and a speed allowing the healthy subjects to execute the movements in a natural way. The task (reaching of the 12 targets) was repeated in a random order three times (in total 36 movements) for each condition, except for the passive modality during day 2, where to avoid muscle fatigue, only one repetition was performed.

### Kinematics recording

During the first session, the kinematics for the free movements was acquired by using a six-camera motion analysis system (Bonita B10, Vicon Oxford Metrics Ltd, Oxford, UK) at 100 Hz. The global reference frame corresponded to the reference frame of the exoskeleton, and it was located at the center of the target panel frame with the *X-axis* medial/lateral pointing to the West target, the *Y-axis* along the vertical direction pointing to the North target, and the *Z-axis* according to the right-hand rule. The markers were selected to model the three degrees of freedom of the shoulder and the flexion/extension of the elbow. Seven markers were placed following the ISB procedure [29]: the processus spinosus of the 7th cervical vertebra (C7), the glenohumeral rotation center (i.e., shoulder acromion), the deepest point of the Incisura Jugularis, the most caudal point on lateral and medial epicondyle for the elbow, the most caudal-lateral point on the radial styloid and the most caudal-medial point on the ulnar styolid for the wrist. Three additional markers were added in the upper arm and one in the midpoint between lateral epicondyle and ulnar styloid process for the calculation of the rotation axis. Finally, three markers were placed on the right hand (metacarpophalangeal joint of the little finger, third finger, and thumb) because in this condition, the subjects held a cylinder with a marker on its top, to mimic the exoskeleton’s handle (Fig. 2a).

In the other sessions and conditions, the joint angles and the position of the EE were acquired by the exoskeleton at 100 Hz.

### Recording of muscle activities

The EMG signals from 16 upper limb muscles (deltoid anterior, DANT, medial, DMED, and posterior, DPOS, pectoralis major, PEC, latissimus dorsi, LAT, infraspinatus, INFRA, rhomboid major, RHO, pronator teres, PRO, biceps brachii short head, BICS, biceps brachii long head, BICL, brachialis, BRA, brachioradialis, BRAD, lateral head of triceps brachii, TRILAT, long head of triceps brachii, TRILONG, superior trapezius, TRAPS, medial trapezius, TRAPM) were recorded by using superficial Ag-AgCl electrodes (Kendall H124SG, ECG electrodes 30 × 24 mm). The skin was cleaned and shaved, and the electrodes were placed, when it was possible, according to the standard procedure for surface electromyography for non-invasive assessment of muscles (SENIAM) guidelines [30].

In order to reduce the variability of the electrode placement among the sessions, the same researcher was in charge of placing the EMG electrodes and the placement of each EMG electrode was every time measured respect to the position of the three closest anatomic landmarks used to place the kinematics markers. The absence of cross-talk among muscles was tested through visual inspection of the EMG signals while performing suitable movements at the moment of the electrode placement.

At the beginning of each session, a manual test for the maximum voluntary contraction (MVC) was performed for each muscle. During the test, subjects were seated and asked to perform isometric contractions with a muscle-specific arm posture against the resistance provided by the researcher (the same researcher across sessions in order to assure measurements’ consistency) [31]. The test was repeated three times for each muscle, with a break after each contraction to prevent muscle fatigue.

### End-effector kinematic analysis

The start and the end points of each center-out or out-center reach were defined as the time points when the speed profile (of the marker placed on top of the cylinder, for free movements, and of the EE of the exoskeleton, for all the other conditions) exceeded or dropped below 2 % of the local maximum value [31]. For the assistive modality during day 2, we used a threshold at the 2 % of the EE trajectory along the main movement direction: in this case, the start and the end points of each center-out or out-center reach were defined as the time points when the EE trajectory changed more than 2 % from the initial position, or when it remained constant in the initial position.

To evaluate the movement execution, three metrics were computed for each target by separately considering the forward and backward movements: the mean distance (**nMD**), defined as the mean absolute distance between the trajectory and the straight line connecting the starting position to the target, normalized to the length of the theoretical path [9]; the **Pace,** defined as the difference between the actual speed and the speed required to follow the metronome tone; and the number of peaks in the speed profile (**nPK**) quantifying the smoothness of the movement [9]. The **nMD**, **Pace**, and **nPK** were computed for the free movements and for the passive modality, since during the assistive modalities the position and the speed of the EE were imposed by the exoskeleton.

For day 3, the point-to-point Euclidian distance (**d**_**EE**_) was computed between passive and assistive modalities with joint and EE control, in order to assess whether the two controls were proposing the trajectories naturally performed by the subjects.

### Joint kinematic analysis

The four angular excursions (SH-Abd, SH-Rot, SH-Flx, and EL-Flx) were extracted directly from the data acquired by the exoskeleton for all the conditions in which the exoskeleton was used.

For free movements, the angular excursions were computed from the kinematic data recorded with the Vicon system. Missed kinematic data were estimated by using cubic spline interpolations, and the data were then low-pass filtered at 10 Hz by using a zero-lag fourth-order Butterworth filter.

Shoulder and elbow joints were modelled as ball-and-socket joints. Their angular excursions were computed with a model accounting for three segments (chest, upper arm, and forearm), where the forearm rotated with respect to the upper arm, which itself rotated with respect to the fixed reference system (i.e., chest), following the Cardan angles convention [32]. SH-Abd corresponded to the shoulder rotation around the *X-axis* (i.e., joint number 1 of the exoskeleton) and it was 0 deg when the humerus was parallel to the trunk with the arm down and 90 deg when the humerus was abducted parallel to the ground. SH-Rot coincided with the shoulder rotation around the *Y-axis* (i.e., joint number 2 of the exoskeleton) and it was 180 deg when the arm was lifted parallel to the ground and extended far from the body and diminished when the arm was rotated in front of the body. SH-Flx equaled the shoulder rotation around the *Z-axis* (i.e., joint number 3 of the exoskeleton) and it was 0 deg when the humerus was parallel to the trunk and 90 deg when the upper arm was lifted forward parallel to the ground. Finally, EL-Flx corresponded to the elbow rotation around the *X-axis* (i.e., joint number 4 of the exoskeleton). It was measured as the angle between the upper arm and the forearm in the transversal plane, and the angle was considered 0 deg when the forearm was perpendicular with respect to the forearm, and it became negative when the forearm was extended.

The kinematic data in each condition were segmented into epochs (i.e., center-out and out-center movement related to each target) and each epoch was time-interpolated over the minimum number of time points across epochs, conditions, and subjects using cubic splines.

The Pearson’s correlation coefficient **(R**_**joint**_) and the absolute distance (**d**_**joint**_) were computed: between the three days in passive modalities to compare the differences in the joint angular excursions across sessions; between free movements and passive modality, to assess whether the use of the exoskeleton modified the joint angular excursions; between assistive and passive modality, to evaluate the performance of passive reaching movements; and for day 3, between passive and assistive modality with joint and EE control, to compare the execution of passive reaching movements by using the two different control strategies.

### EMG signals pre-processing and comparison

The raw EMG signals were detrended in order to eliminate possible bias, high-pass filtered at 50 Hz (Butterworth filter, 7th order) to remove motion artifacts, rectified, low-pass filtered with a cut-off frequency of 10 Hz (Butterworth filter, 7th order) to remove noise, and normalized for the MVC value (i.e., maximum value obtained from the preprocessed data related to the MVC test) of the corresponding session in order to compare the EMG data across subjects and conditions.

In order to compare the muscle activity across conditions the root mean square (**RMS**_**EMG**_) of each muscle was computed after segmentation into epochs (i.e., center-out and out-center movements for each target) and time-interpolation over the minimum number of time points across targets, conditions, and subjects by cubic splines.

### Muscle synergies extraction, ordering, and comparison

For each subject and condition, preprocessed EMG data corresponding to the three repetitions of the 12 forward and backward movements were pooled together and muscle synergies were extracted with the non-negative matrix factorization (NNMF) algorithm [33]. Since the algorithm is iterative, the solution over 50 repetitions explaining the highest overall amount of EMG variance was selected. The NNMF is able to capture the communality of a set of data through the decomposition in a defined number of components, constituted by two coefficients: the weighting coefficients, indicating the involvement of each muscle to each muscle synergy, and the activation coefficients, indicating the timing profile of each muscle synergy.

The number of retained synergies was the minimum number of modules presenting a variance accounted for (VAF) higher than 0.95 [34]. For each condition, the same number of muscle synergies was retained for all subjects to allow an easy intra-group comparison.

To simplify the visualization and the comparison, the weighting and the activation coefficients of the muscle synergies were matched among the different subjects and conditions according to the maximum similarity of the weighting coefficients measured by using the scalar products (**DOT**_**SYN**_).

Finally, in order to compare the level of activity of each muscle synergy across the different conditions, the root mean squared value (**RMS**_**SYN**_) of the activation coefficients was computed after epochs segmentation (i.e., center-out and out-center movements for each target) and time-interpolation over the minimum number of time points across epochs, conditions, and subjects by cubic splines.

### Estimation of spatiotemporal motoneuronal activity

Pre-processed EMG signals were used to estimate the MN activity in the spinal cord, as previously described in literature to investigate the muscle activity in the lower extremities [23, 24, 35–37].

For each spinal segment, the MN activity during forward and backward movement was computed as the weighted summation of all the normalized EMG waveforms obtained from the muscles innervated by such segment [22, 24]. The weight coefficients approximating the rostro-caudal distribution of the MN pools innervating the upper limb muscles included in the study were located in the segments from C2 to T1 as reported by Kendall [38, 39] (see Table 2). For each condition the MN activity was averaged over subjects and repetitions. The Center of Activity (**CoA**) was computed for each time frame as the centroid of the activity of the spinal maps [24].

**Table 2:** Mapping of the muscle activity on the spinal cord segments

	DANT	PEC	LAT	INFRA	RHO	PRO	BICS	BICL	BRA	BRAD	TRILAT	TRILONG	DMED	DPOS	TRAPS	TRAPM
C2															X	X
C3															X	X
C4				X	X										X	X
C5	X	X		X	X		X	X	X	X			X	X		
C6	X	X	X	X		X	X	X	X	X	X	X	X	X		
C7		X	X			X					X	X				
C8			X								X	X				
T1											X	X				

X corresponds to a weight coefficient of 1

For representative purposes and to simplify the comparison of the muscle synergies, we also computed the spatiotemporal organization of the muscle synergies [24]. For each muscle synergy, the MN activity was calculated as above from a putative EMG dataset that was obtained by combining the synergy’s timing activation vector with the correspondent weighting coefficient vector.

In order to assess the similarity between two different spinal maps, we used the 2D correlation coefficient between the two maps (**R**_**Map-EMG**_ for EMG and **R**_**Map-SYN**_ for muscle synergies) [37] and the mean distance (**d**_**COA**_) of their related **CoA**s [24]. The **R**_**Map-EMG**_, the **d**_**COA**_, and the **R**_**Map-SYN**_ were computed: between free movements and passive modality; between assistive and passive modality; and for day 3, between passive and assistive modality with joint and EE control.

### Statistics

The statistical analyses were performed by using custom routines written in Matlab environment (Mathworks Inc., Natick, MA, USA) comparing the conditions as reported in Table 3.

**Table 3:** Design of the analysis

	EE kinematics	Joint kinematics	EMG activity	Muscle synergies	Spinal maps
Passive day 1 vs day 2 vs day 3	**nMD**, **nPK**	**R**_**joint,**_ **d**_**joint**_	**RMS**_**EMG**_, MVC	-	-
Free movements vs passive day 1	**nMD**, **Pace**, **nPK**	**R**_**joint**,_ **d**_**joint**_	**RMS**_**EMG**_	**RMS**_**SYN**,_ weighting coefficients, **R**_**Map-SYN**_	**R**_**Map-EMG**_, **d**_**CoA**_
Passive day 1 vs assistive	**-**	**R**_**joint**,_ **d**_**joint**_	**RMS**_**EMG**_	**RMS**_**SYN**,_ weighting coefficients, **R**_**Map-SYN**_	**R**_**Map-EMG**_, **d**_**CoA**_
Passive day 3 vs assistive with EE control, passive day 1 vs assistive with joint control	**d**_**EE**_	**R**_**joint**,_ **d**_**joint**_	**RMS**_**EMG**_	**RMS**_**SYN**,_ weighting coefficients, **R**_**Map-SYN**_	**R**_**Map-EMG**_, **d**_**CoA**_

The metrics (bold) used to assess: the variability across sessions (passive modality day 1 vs day 2 vs day 3), ALEx transparency (free movements vs passive modality day 1), the execution of active and passive movements (passive modality day 1 vs assistive modality), and the control at the EE and at the joints (passive modality day 3 vs assistive modality with EE control and passive modality day 3 vs assistive modality with joint control)

For each metric, comparisons were computed mainly between two conditions, thus the main test used for the analysis was the Wilcoxon signed-rank test (α = 0.05) for each reaching direction. P-values were Bonferroni corrected for the number of comparisons (i.e., number of directions) in order to increase the reliability of our results. A Kruskal-Wallis test (α = 0.05) was used to compare the passive conditions among the three sessions.

## Results

### The passive condition was comparable and repeatable across the three sessions

In order to assess the inter-session variability, we compared the MVC values acquired in each of the three sessions, and the kinematics and the muscle activity related to the three conditions in which the exoskeleton was used in passive modality. For this purpose, one repetition was chosen for day 1 and day 3. Differences were tested among the three conditions with a Kruskal-Wallis test (α = 0.05) for each subject that was recorded in all the three sessions, and a Wilcoxon signed-rank test (α = 0.05) for the six subjects that were recorded in day 1 and day 2.

Tables 4 and 5 report the mean MVC values for all the subjects in the first and second session and for the three subjects participating to the three sessions, respectively. No significant differences were found in the MVC values between the first and the second session, and across the three sessions.

**Table 4:** MVC values for each muscle in the first and second session

	DANT	PEC	LAT	INFRA	RHO	PRO	BICS	BICL	BRA	BRAD	TRILAT	TRILONG	DMED	DPOS	TRAPS	TRAPM
S1	0.46 (0.09)	0.31 (0.06)	0.26 (0.07)	0.29 (0.04)	0.42 (0.06)	0.38 (0.05)	0.22 (0.06)	0.40 (0.06)	0.24 (0.04)	0.29 (0.04)	0.36 (0.09)	0.38 (0.08)	0.34 (0.06)	0.38 (0.10)	0.31 (0.07)	0.30 (0.07)
S2	0.41 (0.05)	0.30 (0.05)	0.29 (0.07)	0.30 (0.07)	0.28 (0.04)	0.31 (0.12)	0.44 (0.07)	0.46 (0.08)	0.18 (0.02)	0.43 (0.12)	0.26 (0.03)	0.32 (0.06)	0.49 (0.04)	0.52 (0.07)	0.35 (0.07)	0.35 (0.05)

S1 is for session 1 and S2 is for session 2. The table reports the mean and the standard error (in brackets) among six participants. No significant differences were found between the MVC values of the two sessions

**Table 5:** MVC values for each muscle and for the three sessions

	DANT	PEC	LAT	INFRA	RHO	PRO	BICS	BICL	BRA	BRAD	TRILAT	TRILONG	DMED	DPOS	TRAPS	TRAPM
S1	0.53 (0.20)	0.33 (0.07)	0.26 (0.12)	0.33 (0.10)	0.40 (0.06)	0.43 (0.12)	0.22 (0.13)	0.41 (0.06)	0.28 (0.11)	0.34 (0.03)	0.49 (0.16)	0.49 (0.16)	0.39 (0.07)	0.34 (0.04)	0.41 (0.16)	0.31 (0.13)
S2	0.33 (0.05)	0.32 (0.08)	0.30 (0.14)	0.34 (0.14)	0.32 (0.08)	0.16 (0.05)	0.38 (0.05)	0.42 (0.11)	0.19 (0.04)	0.47 (0.32)	0.28 (0.08)	0.35 (0.12)	0.52 (0.12)	0.57 (0.17)	0.40 (0.16)	0.44 (0.01)
S3	0.38 (0.13)	0.21 (0.04)	0.15 (0.03)	0.25 (0.05)	0.27 (0.04)	0.16 (0.02)	0.25 (0.04)	0.41 (0.08)	0.10 (0.02)	0.33 (0.02)	0.27 (0.04)	0.29 (0.15)	0.45 (0.07)	0.36 (0.08)	0.30 (0.07)	0.36 (0.05)

S1 is for session 1, S2 is for session 2, and S3 for session 3. The table reports the mean and the standard error (in brackets) among three healthy participants. No significant differences were found among the MVC values of the three sessions

**Fig. 3:**
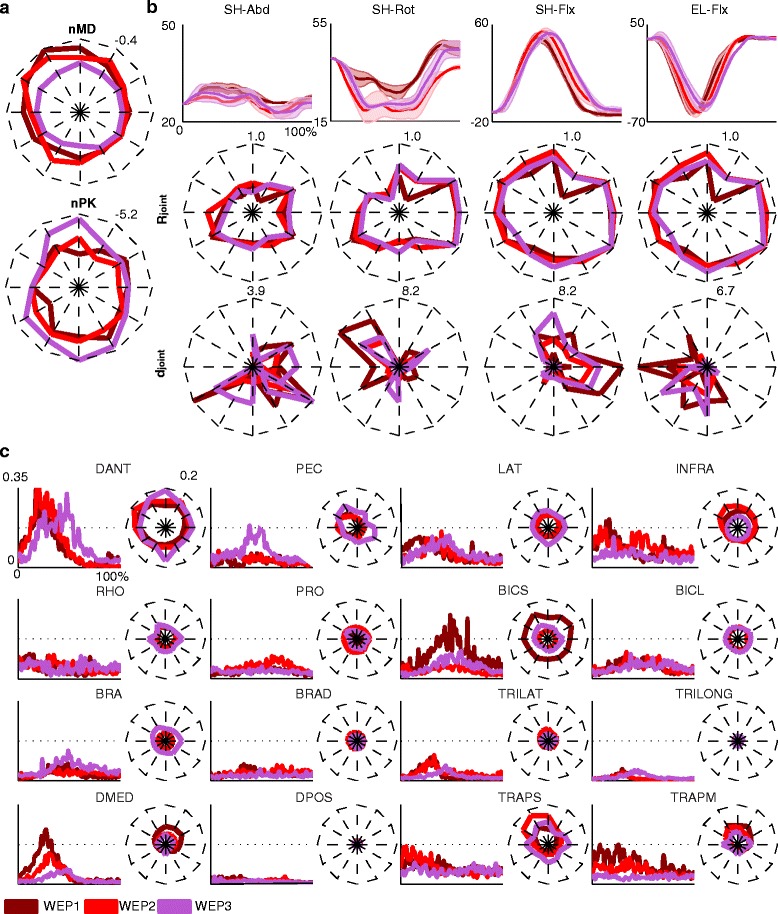
Movement execution and muscle activity across the three sessions, while performing the reaching tasks wearing the exoskeleton in passive modality (day 1 (WEP1), day 2 (WEP2), and day 3 (WEP3)). **a** The end-effector parameters (*i.e.*, the mean distance, **nMD**, and the numbers of peaks in the speed profile, **nPK**) averaged across the three subjects for day 1, day 2, and day 3, are represented for the twelve targets arranged in a clock-like fashion (each value represents the mean across the subjects for the forward and backward movements) in one repetition. Dark red, light red, and purple lines code day 1, day 2, and day 3, respectively. The maximum value for the **nMD** and **nPK** is reported in the upper right corner of each plot. **b** The averaged four angular trajectories (SH-Abd, SH-Rot, SH-Flx, and EL-Flx) are reported for a representative target (North). The mean and the standard errors refer to the three subjects and one repetition. On the x-axis, the duration of the movement is represented in percentage and it includes the forward and backward movement. On the bottom, the Pearson correlation coefficients (**R**_**joint**_) and the angular distance (**d**_**joint**_) in deg are reported for each target. Each value represents the mean across the three subjects. Dark red lines code the average correlation between the day 1 and day 2 and between day 1 and day 3, light red lines code the average correlation between day 2 and day 1 and between day 2 and day 3, and purple lines code the average correlation between day 3 and day 1 and between day 3 and day 2. The maximum value for the distance and the correlation is reported in the upper right corner of each plot. **c** The muscle activity for a representative target (*North*) averaged across the three subjects for the three days. On the x-axis the duration of the movement is represented in percentage, and it includes the forward and backward movement. The **RMS**_**EMG**_ for the twelve targets is also reported for each muscle. Dark red, light red, and purple lines code day 1, day 2, and day 3, respectively. The maximum value for the **RMS**_**EMG**_ is reported in the upper right corner of the DANT muscle

Additionally, no significant differences were found in the performance of the movements across the three days (Fig. 3a) and between day 1 and day 2 (data not showed) for the **nMD** and the **nPK**. The joint angular excursions were also very similar in the three sessions (mean **R**_**joint**_ = 0.69 and mean **d**_**joint**_ = 41 deg for the four joint angular excursion and the twelve targets, Fig. 3b)_**.**_

The timing and the level of the muscle activity were generally preserved among the three sessions for all muscles (average **RMS**_**EMG**_ difference across sessions and muscles: 0.03). Some exceptions were constituted by BICS and DMED that showed a higher activity in day 1 (Fig. 3c).

Overall, the results show that the movement execution and the muscle activity were very similar for the three days; consequently, the results can be reasonably compared across the different sessions. In this regard, in the following analysis, we used the movements performed in passive modality in day 1 as comparison for the free movements and the assistive modality and the movements performed in passive modality in day 3 for assistive joint and EE controls.

### The performance during reaching was similar in passive and assistive modalities but it slightly differed from natural free reaching

**Fig. 4:**
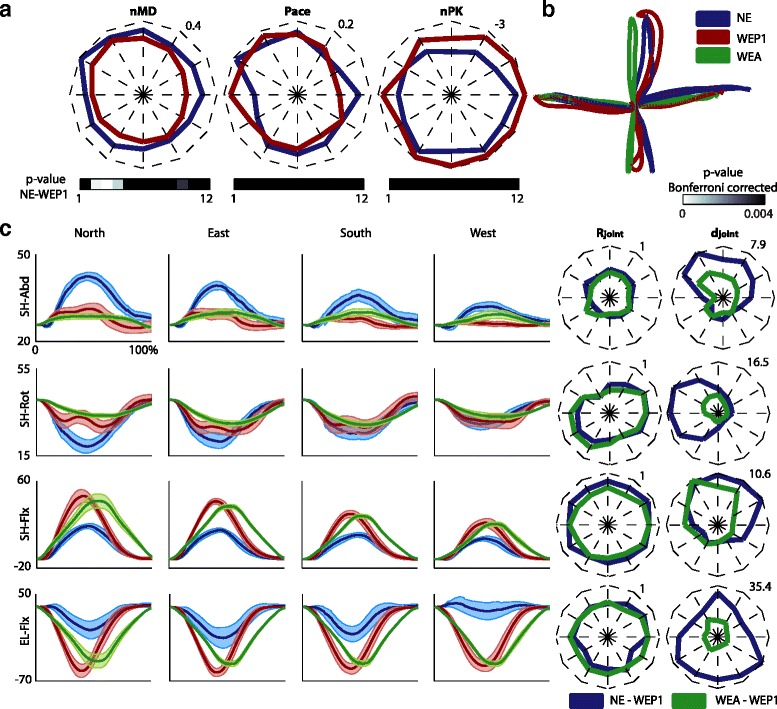
The execution of the reaching task in the conditions: free movements (NE), passive modality day 1 (WEP1), and assistive modality (WEA). **a** Averaged mean distance (**nMD**), pace (**Pace**), and numbers of peaks in the speed profile (**nPK**) across the six subjects are represented for the twelve targets arranged in a clock-like fashion (each value represents the mean across six subjects and three repetitions for the forward and backward movements). Dark blue and red lines code the free movements and the passive modality, respectively. The maximum value for the **nMD**, **Pace**, and **nPK** is reported in the upper right corner of each plot. On the bottom, the p-values, Bonferroni corrected for the number of targets, related to the comparison of free movements and passive modality are reported in a gray scale for each target. **b** The averaged EE trajectories for the free movements (*blue line*), the passive modality (*dark red line*), and the assistive modality (*green line*) for four targets: North, East, South, and West. **c** The averaged four angular trajectories (Sh-Abd, SH-Rot, SH-Flx, and EL-Flx) are represented for four representative targets (North in the first column, East in the second column, South in the third column, and West in the fourth column). The mean and the standard errors refer to six subjects and three repetitions. On the x-axis the duration of the movement is represented in percentage, and it includes the forward and backward movement. Blue, dark red, and green lines code the free movements, the passive modality, and the assistive modality, respectively. On the right, the Pearson correlation coefficients (**R**_**joint**_ in the first column) and the angular distance (**d**_**joint**_ in the second column) in deg are reported for each target. Each value represents the mean across six subjects and three repetitions. Blue lines code **R**_**joint**_ and **d**_**joint**_ between free movements and passive modality. Green lines code **R**_**joint**_ and **d**_**joint**_ between passive and assistive modality. The maximum value for **R**_**joint**_ and **d**_**joint**_ is reported in the upper right corner of each plot. Figure 4a was already reported in [21]

The EE kinematics during the reaching tasks was slightly modified by using the exoskeleton (Fig. 4a, already reported in [21]). The accuracy (**nMD**) was significantly higher when wearing the exoskeleton in passive modality with respect to free movements in particular for the East targets (*p* < 0.003). The **Pace** and the **nPK**, instead, were comparable between the two conditions, albeit free movements showed a trend of higher smoothness (**nPK**).

The joint angular excursions were very similar between passive and assistive modality, but they differed from the condition without the exoskeleton (Fig. 4b): while the amplitude of the angular excursions was modified, particularly for the North and South targets, the dynamics of the movement was generally maintained. Indeed, only SH-Abd was not modulated in the conditions with the exoskeleton (**R**_**joint**_ between free movements and passive modality for SH-Abd was generally lower than the **R**_**joint**_ for SH-Rot, SH-Flx, and EL-Flx). Instead, the abduction of the shoulder, especially for the targets in the North direction (see **d**_**joint**_ of SH-abd in targets 1, 2, 10, 11, and 12), and the rotation of the shoulder for the targets in the West direction (see **d**_**joint**_ of SH-rot in targets 8, 9, and 10) were reduced. Moreover, wearing the exoskeleton increased the flexion-extension of the shoulder for the targets in the North direction (see **d**_**joint**_ of SH-flx in targets 1, 2, 11, and 12), and the flexion of the elbow, especially for the target in the North and South direction (see **d**_**joint**_ of EL-Flx in targets 4, 5, 6, 7, 8, and 12).

Concerning the differences between active and passive movements with ALEx, it is possible to notice that in the assistive modality the SH-Abd and SH-Rot tended to be stabilized with a reduced angular excursion, while the movements were mainly achieved with the modulation of the flexion-extension of the shoulder and elbow. Finally, it is also possible to notice that the subjects in the passive modality tended to anticipate the maximum extension-flexion of the upper limb (see SH-Flx and EL-Flx) with respect to the movements controlled by ALEx.

### Muscle activity was generally preserved using the exoskeleton but it was reduced when wearing ALEx in assistive modality

**Fig. 5:**
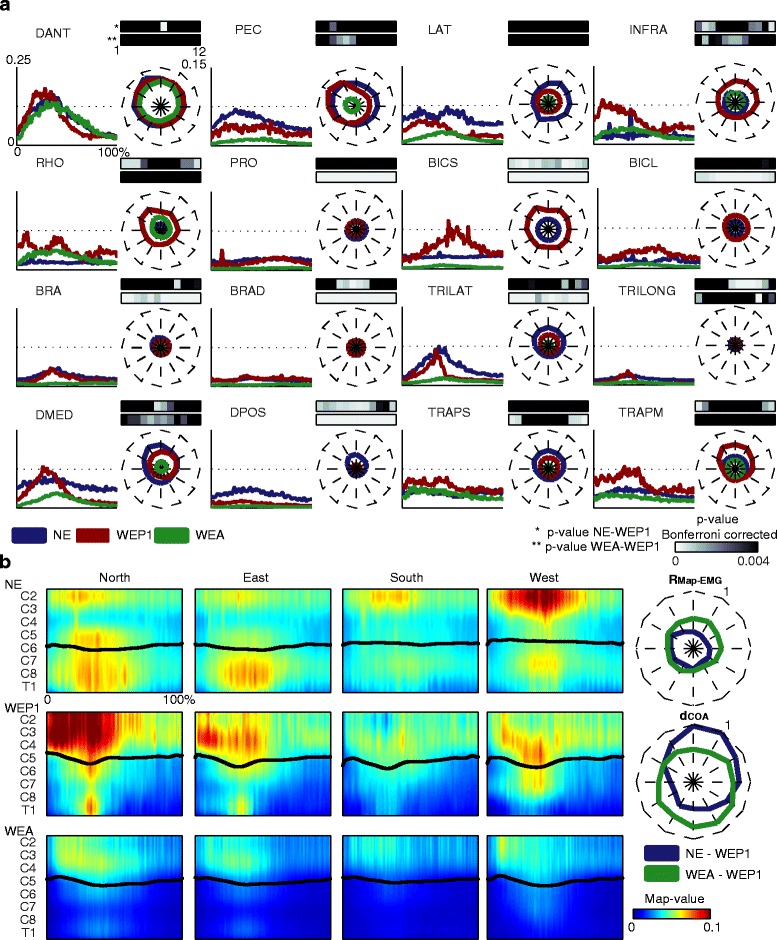
The muscle activity in the conditions: free movements (NE), passive modality day 1 (WEP1), and assistive modality (WEA). **a** The pre-processed EMG signals for a representative target (*North*) in the three conditions. The mean values refer to six subjects and three repetitions. The duration of each movement is represented in percentage, and it includes the forward and backward movement. In correspondence to each muscle activation is reported also the **RMS**_**EMG**_ for the 12 targets. Blue, dark red, and green lines code the free movements, the passive modality, and the assistive modality, respectively. The maximum value for the **RMS**_**EMG**_ is reported in the upper right corner of the DANT muscle. On top of the **RMS**_**EMG**_ plot, the p-values, Bonferroni corrected for the number of targets, between free movements and passive modality (*top row*) and between passive and assistive modality (*bottom row*) for the twelve targets are reported according to a gray scale. **b** The spinal maps for four representative targets (North in the first column, East in the second column, South in the third column, and West in the fourth column): in the first row, the free movements; in the central row, the passive modality; in the bottom row, the assistive modality. Each spinal map is the average among the maps of six subjects and three repetitions. On the x-axis the duration of the movement is represented in percentage and it includes the forward and backward movement. The black lines represent the averaged **CoA**. On the right, the 2D correlation coefficients (**R**_**Map-EMG**_) and the mean distance (**d**_**COA**_) of the related **CoAs** are reported for each target. Dark blue lines code the comparison between free movements and passive modality. Dark green lines code the comparison between passive and assistive modality. The maximum value for the correlation and the distance is reported in the upper right corner of each plot

During free movements and movements with the exoskeleton in passive and assistive modality, the most active muscles were the postural back muscles (LAT, INFRA, and RHO), the shoulder elevators (TRAPS and TRAPM), the shoulder flexor (DANT) and abductor (DMED), the PEC, and the TRILAT (see Fig. 5a). The muscles of the back (INFRA and RHO), the shoulder’s muscles (DANT and DMED), and TRILAT showed a main peak during the forward movement towards the targets. The LAT, in free movements, and the shoulder elevator muscles, in both conditions (free movements and passive modality), were active during the whole reaching movement. The timing of muscle activity was generally preserved among the three conditions, particularly for DANT, PEC, and TRILAT muscles.

As already reported in [21], wearing the exoskeleton induced a redistribution of muscle contribution for the execution of the reaching task: the control of the shoulder and of the elbow extension in free movements was substituted by the control of the back muscles and of the elbow flexors. In particular, during free movements, DMED (*p* < 0.003) for the South targets and DPOS (*p* < 0.004) for all directions showed a higher activity. Whereas in passive modality the following muscles had a stronger activity: INFRA (*p* < 0.002) and RHO (*p* < 0.003) in particular for the North and the West targets, and BICS (*p* < 0.001) for all directions.

By using ALEx in assistive modality, the muscles were activated similarly than in passive modality, but the level of activity of most of the muscles (PRO, BICS, BICL, BRA, BRAD, TRILAT, and DMED) was significantly lower than in the passive modality for all the directions (*p* < 0.004).

The spinal maps for free movements were characterized by a main period of activation between the 10 % and the 60 % of the reaching task (see Fig. 5b). The higher activity was located in the lower cervical and in the upper thoracic segments for the targets North and East, and primarily in the cervical and less in the upper thoracic segments for the South and West targets. The spinal maps when wearing the exoskeleton in passive modality differed from those of free movements (mean **R**_**Map-EMG**_ for the 12 targets: 0.33). In particular, there was a shift of activity to upper cervical locations when using ALEx for the North and East targets (the mean **d**_**COA**_ was 0.91 and 0.77 for the North and the East target, respectively). Indeed, the activity for North and East targets in passive modality was more similar to the one in the West target for free movements, with a higher involvement of the C5 and C6 segments, while the activity in the South and West directions had a similar location but it was attenuated.

Finally, when wearing the exoskeleton in assistive modality, the MN activity was generally similar, but less intense with respect to the passive modality (mean **R**_**Map-EMG**_ for the 12 targets: 0.47).

### Muscle coordination was modified across natural movements and movements executed with ALEx in *passive* and assistive modality

**Fig. 6:**
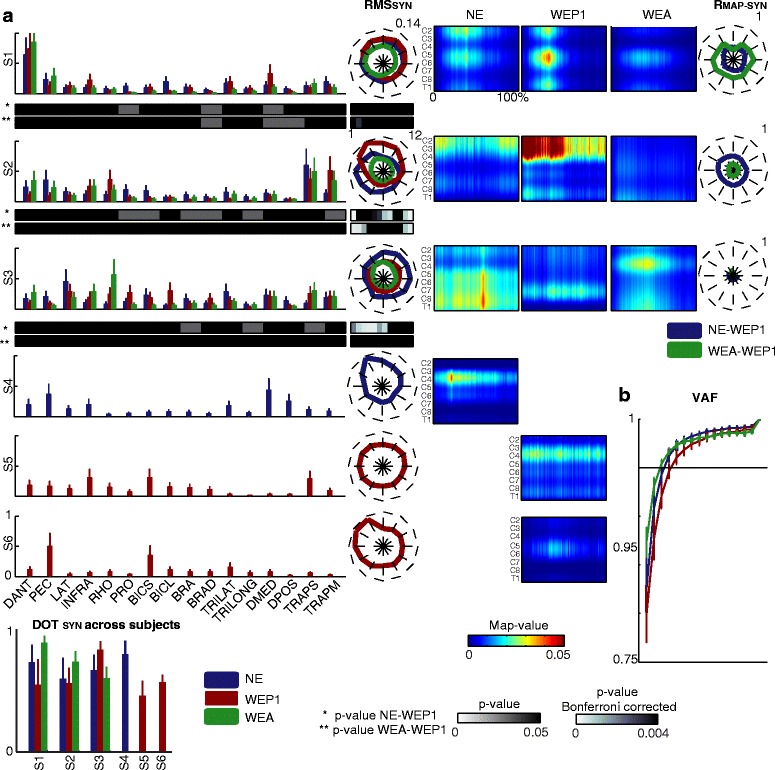
The muscle synergies in the conditions: free movements (NE), passive modality day 1 (WEP1), and assistive modality (WEA). **a** In the first column, the weighting coefficients of the muscle synergies are represented for the three conditions (blue for free movements; dark red for passive modality; and green for assistive modality). The mean and standard errors refer to six subjects. Below S1, S2, and S3, the p-values for the weighting coefficients between free movements and passive modality (*top row*) and between passive and assistive modality (*bottom row*) are reported using a gray scale. In the second column the **RMS**_**SYN**_ is reported for the 12 targets. The **RMS**_**SYN**_ corresponds to the average values across six subjects and three repetitions. Blue, dark red, and green lines code the free movements, the passive modality, and the assistive modality, respectively. The maximum value for the **RMS**_**SYN**_ is reported in the upper right corner of the plot of S1. On the bottom of the **RMS**_**SYN**_ the p-values, Bonferroni corrected for the number of targets, between free movements and passive modality (*top row*) and between passive and assistive modality (*bottom row*) are reported using a gray scale**.** On the bottom of the weighting coefficients, the average DOT_SYN_ for each synergy for the three conditions (blue for free movements, dark red for passive modality, and green for assistive modality). The mean and standard errors refer to six subjects. On the left, the spatiotemporal organization maps for each muscle synergy in free movements (*first column*), passive modality (*second column*), and assistive modality (*third column*). Each map is the average one among six subjects and three repetitions. On the x-axis the duration of the movement is represented in percentage and it includes the forward and backward movement. In the fourth column, the **R**_**Map-SYN**_ are reported for all the targets. Blue lines code the comparison between free movements and passive modality. Green lines code the comparison between passive and assistive modality. The maximum value for the correlation is reported in the upper right corner of each plot. **b** The average VAF for the three conditions (blue for free movements, dark red for passive modality, and green for assistive modality)

Muscle synergies analysis was performed to assess possible effects of the use of the exoskeleton on muscle coordination in passive and assistive modality (Fig. 6).

Four muscle synergies, similar to those reported in our previous work [21], were extracted for each subject for free movements (3.5 ± 1.4); whereas five muscle synergies were found when wearing the exoskeleton in passive modality (4.6 ± 1.5) and three in assistive modality (3.2 ± 0.8) according to the criterion of the VAF >0.95. Muscle synergies were similar across subjects (mean **DOT**_**SYN**_ was 0.70, 0.59, and 0.74 for free movements, passive modality, and assistive modality, respectively).

During free movements, muscle synergies were similar to those already reported in literature for an analogous task [31]. In particular, S1 mainly grouped the muscles dedicated to the flexion-extension and abduction-adduction of the shoulder (*i.e.,* DANT and DMED) and it was mainly active from the 20 % to the 50 % of the movement (*i.e.,* in the forward reaching toward the target). Additionally, S1 was primarily located in C2, C5-C6, and T1. S2 accounted for the muscles responsible for the shoulder elevation (i.e., TRAPS and TRAPM), it was active during the whole movement, and it was characterized by a burst of activity in the upper cervical segments (C2 and C3). S3 involved the back and postural muscles, in particular LAT, RHO, INFRA, DMED, TRAPS, and TRAPM; it was active during the whole movement with a higher burst of activity near the reaching of the target (between 20 and 70 % of the reaching), when the limb reached its maximum extension. Moreover, it was mainly located between C6 and C8. Finally, S4 grouped DMED, DPOS, and PEC. It was mainly active in the forward movement, in particular on the midway between the starting and the target position, and it was mainly located in C3 and C4.

Three muscle synergies (S1 –S3) were common between movements performed without and with the exoskeleton in passive and assistive modality (**DOT**_**SYN**_ >0.48): the involvement of each muscle in the shared muscle synergies was very similar, and those presenting a significant difference across conditions (*p* < 0.05) were characterized by a weighting coefficient usually lower than 0.3 [40]. Despite the preservation of the structure, the spatiotemporal activation of the shared muscle synergy changed across conditions.

S1 was characterized by three bursts of activity with a similar intensity and located in the same segments for free movements and passive modality, while in the assistive modality the level of activity was slightly lower. The activity of S2 was higher at the beginning and at the end of the movement for free movements, especially for the targets near the South-West direction, while it was more intense in the forward movement for passive modality in the targets near the North direction (*p* < 0.004). As for S1, the activity of S2 in the assistive modality was lower in particular for the North targets (*p* < 0.001). The spatiotemporal organization of S3 highly varied across conditions (the average **R**_**Map-SYN**_ over the 12 targets was 0.11 between free movements and passive modality, and 0.04 between assistive and passive modality). Indeed, in the passive modality, the burst of activity was localized between C7 and C8, it was continuous for the whole movement duration, and it was significant lower than in free movements in particular for the East targets (*p* < 0.002). Whereas, in the assistive modality, it was mainly located in C3, C4, and T1, and it occurred from the 10 % to the 60 % of the movement.

Finally, wearing the exoskeleton in the passive modality favored the activation of two additional muscle synergies (S5 and S6) that substituted the activation of S4. In particular, S5 accounted for the activity of INFRA, BICS, and TRAPS, it was active during the whole movement with higher peaks during the forward movement, and it was mainly located in C3 and C4. S6 grouped PEC and BICS. It was active at the end of the forward movement (from the 30 % to the 50 % of the movement) with a higher activity for the West-North direction and localization in C5-C6.

### Muscle coordination elicited by the two active control strategies was similar to active movements especially for the joint control

The statistical analysis did not show any significant difference in the movement execution and in the muscle activity and coordination between assistive modality with joint and EE control, but a trend and some differences were evident between the two conditions.

**Fig. 7:**
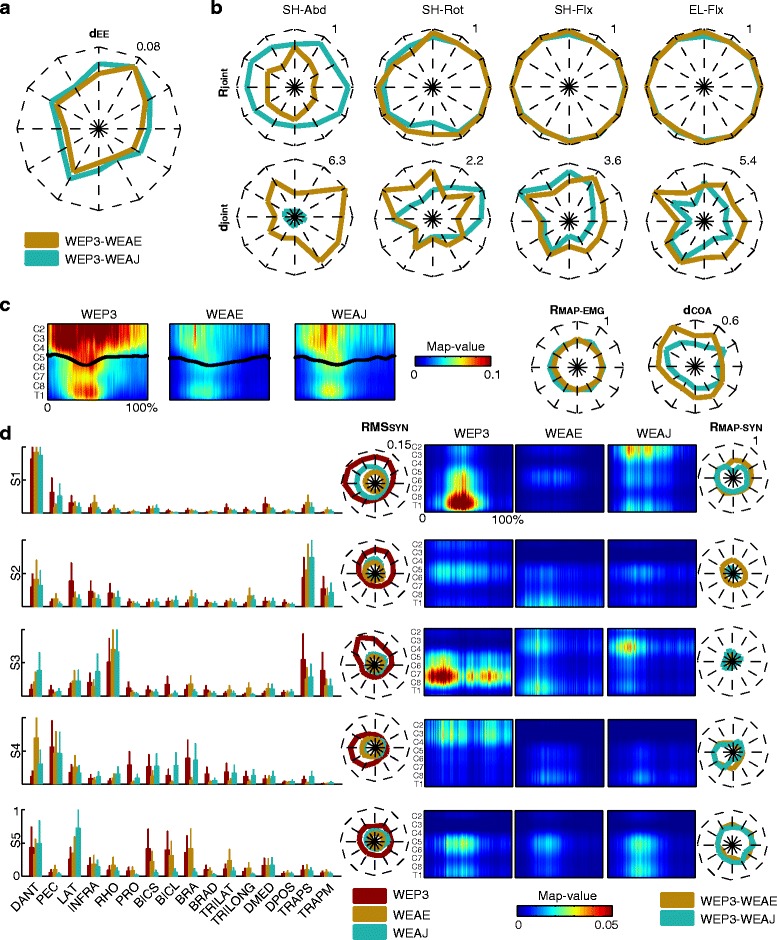
The movement execution and muscle activity in the third session. **a** Averaged point-to-point Euclidean distance (**d**_**EE**_) for the EE trajectories between passive (WEP3) and assistive modality with EE control (WEAE - dark cyan line) and between passive and assistive modality with joint control (WEAJ - dark yellow line) for the twelve targets arranged in a clock-like fashion. The mean values refer to three subjects and three repetitions. **b** Mean Pearson correlation coefficients (**R**_**joint**_) and the mean angular distance (**d**_**joint**_) in deg for the twelve targets arranged in a clock-like fashion (each mean value refers to three subjects and three repetitions). Dark cyan lines code the **R**_**joint**_ and **d**_**joint**_ between passive and assistive modality with joint control. Dark yellow lines code the **R**_**joint**_ and **d**_**joint**_ between passive and assistive modality with EE control. The maximum value for the correlation and for the distance is reported in the upper right corner of each plot. **c** Spinal maps for a representative target (i.e., North target) for the three conditions (left: passive modality; center: assistive modality with EE control; right: assistive modality with joint control). On the x-axis the duration of the movement is represented in percentage and it includes the forward and backward movement. The black lines code the mean **CoA**. The mean values for the spinal maps and the **CoA** refer to three subjects and three repetitions. On the right, the 2D correlation coefficient between two maps (**R**_**Map-EMG**_) and the mean distance (**d**_**COA**_) of the related **CoAs** are reported. Dark cyan lines code **R**_**Map-EMG**_ and **d**_**COA**_ between passive and assistive modality with joint control. Dark yellow lines code **R**_**Map-EMG**_ and **d**_**COA**_ between passive and assistive modality with EE control. The maximum value for the correlation and the distance is reported in the upper right corner of each plot. **d** In the first column, the weighting coefficients are represented for the three conditions (in dark red: passive modality; in dark cyan: assistive modality with joint control, in dark yellow: assistive modality with EE control). The mean and standard errors refer to three subjects. In the second column, the **RMS**_**SYN**_ is reported for the 12 targets and it corresponds to the average values across three subjects and three repetitions. Dark red, dark cyan, dark yellow lines code the passive modality, the assistive modality with joint control, and the assistive modality with EE control, respectively. The maximum value for the **RMS**_**SYN**_ is reported in the upper right corner of the plot of S1. In the third column, the spatiotemporal organization of each muscle synergies for passive modality (*first column*), assistive modality with EE control (*second column*), and assistive modality with joint control (*third column*) is reported for target North. Each map is the average among the maps of three subjects and three repetitions. On the x-axis the duration of the movement is represented in percentage and it includes the forward and backward movement. In the fourth column, the **R**_**Map-SYN**_ for all the targets are reported. Dark cyan lines code the comparison between passive and assistive modality with joint control. Dark yellow lines code the comparison between passive and assistive modality with EE control. The maximum value for the correlation is reported in the upper right corner of S1

As expected, the control at the joints was generally more precise in reproducing trajectories at the joints than the control at the EE, in particular for the shoulder abduction-adduction (**R**_**joint**_ **>** 0.88 and **d**_**joint**_ < 1.67 deg between passive and assistive modality with joint control for SH-Abd, SH-Rot, SH-Flx, and EL-Flx in Fig. 7b), while the control at the EE allowed more precise trajectories at the EE (see the lower **d**_**EE**_ between passive and assistive modality with EE control in Fig. 7a).

As expected, the level of activity of the muscles in the two active modalities was lower for most of the muscles respect than the passive modality. However, the control at the joints promoted a slightly higher muscle activity than the control at the EE in all muscles (data not showed). Moreover, the overall upper limb muscle activity for the joint control resulted more similar to that for the passive modality than that for the EE control (see the higher **R**_**Map-EMG**_ and the lower **d**_**COA**_ between passive and assistive modality with joint control in Fig. 7c).

For an easy inter-group comparison, five muscle synergies were retained for the passive modality. Five muscle synergies were also found for assistive modality with EE control (4.7 ± 0.6) and joint control (5 ± 1) according to the criterion of VAF >0.95 (Fig. 7d). Muscle synergies were similar across subjects (mean **DOT**_**SYN**_ was 0.78 for passive and assistive modality with joint control, and 0.71 for assistive modality with EE control, data not showed).

The five muscle synergies extracted in passive modality corresponded to the five synergies found for day 1 (**DOT**_**SYN**_ > 0.49), and they were also similar to the five synergies extracted for EE and joint control (**DOT**_**SYN**_ > 0.48 between passive and assistive modality with EE control, and **DOT**_**SYN**_ > 0.55 between passive and assistive modality with joint control).

The level of activation of all the muscle synergies was lower for both assistive modalities with respect to passive modality, but S1, S3, and S5 showed a higher activity for the joint control (see **RMS**_**SYN**_, Fig. 7d). Moreover, the spatiotemporal organization of the muscle synergies differed between passive and assistive modality, except for S5 (average **R**_**MAP-SYN**_ over the 12 targets: 0.5), but it was similar between the two control strategies.

## Discussion

In this work, we firstly extended our previous work [21] on the evaluation of ALEx analyzing joint kinematics and spinal maps and including statistical analysis for all movement directions, in order to deeper assess its transparency and its application as a rehabilitative tool. In this regard, we consider that the robot behaves transparently if movement execution with and without the robot show kinematically equivalent EE and joint trajectories and similar patterns of muscle activation and coordination.

Then, we were also interested in investigating the effects of different rehabilitative strategies and exercises yielded by the exoskeleton, in order to provide more insights on the use of robots for rehabilitation. In this regard, accordingly to previous findings on the effect of gravity compensation on upper limb muscle activity [31, 41], we hypothesize that passive movements elicit a low but still coordinated muscle activity, analogously to what happens during fully supported upper limb movements.

Our results generally confirmed our hypothesis and, interestingly, we found that the choice of the desired trajectories for passive training may influence the inferred muscle organization.

### Evaluation of muscle coordination and organization by using muscle synergies and spinal maps

Muscle synergies obtained from the factorization of EMG signals collected during the performance of different motor tasks have been recently proposed by many authors to study muscle coordination and motor control [40, 42, 43]. Indeed, the combination of few muscle synergies can explain the main spatiotemporal characteristics of muscle activation during movements [44, 45]. Moreover, the analysis of muscle synergies has also been proposed in rehabilitation to highlight the modifications of motor control due to several neural pathologies [46].

The muscle synergies obtained in our analysis generally agree with the literature that report from 2 to 8 muscle synergies during upper limb movements [40, 44, 45, 47–49], but they offer a more compact description of the variability of the EMG signals respect than that already found by previous authors including similar muscle groups and adopting an equivalent motor task [31, 50].

So far, spinal maps have been characterized mainly in lower limb motor tasks [22–25], and, for the best of our knowledge, no previous works have studied spinal maps during reaching movements. We proposed this method since we believe that it represents a useful tool to explore muscle organization also for upper extremities, where the muscular timing activation is often more complex. The analysis of the MN activity offers the possibility to assess if different factors (such as the use of the exoskeleton or the different modalities of control) would have an impact on the spinal cord activity. In addition, in this work we proposed a combination of the two methodologies, i.e., the spatio-temporal organization of muscle synergies, which may provide an immediate description of each muscle synergy and of the location and of the dynamics of the spinal circuitries eliciting the activation of the synergy. This approach has never been adopted so far, but it has been suggested by the spinal distribution of the Gaussian activation components estimating the timing activation of the muscle synergies, proposed by Ivanenko and colleagues [24].

Our results show that these three methodologies are meaningful to explore the information provided by the EMG signals and they were able to pinpoint higher differences across conditions than by looking at the features of the EMG envelops. In fact, muscle synergies and their spatio-temporal organization proved to be sensitive to the biomechanical request of the task, *i.e.* to the use of the exoskeleton, and to the proposed trajectories (straight lines or natural ones). While spinal maps were mainly sensitive to the variations of the level of the muscle activity across conditions, in particular between passive and assisted movements.

### Consistency of the results across sessions

Recordings were performed in three different sessions, in order to avoid muscle fatigue and adaptation to the device. However, since differences in subjects’ postures could introduce variability in the kinematic and muscle activity, we assessed the inter-session variability and the repeatability of the assistance of ALEx over time.

No significant differences were found in the performance of the movements across sessions, as shown by the EE trajectories and the joint angular excursions, and in the muscle activity, as shown by the preserved timing and level of activity. Only few differences, which could be ascribable to a slight variation in the electrode placement, were found for the level of activity on the first session for BICS and DMED.

Overall, the repeatability of the kinematics and muscle activity across sessions may be favored by the easy and controlled setup of ALEx. As a matter of fact, the preparation of the subject consisted only in the alignment of the user’s shoulder acromion to the center of rotation of ALEx’s shoulder joint, which could be easily performed by regulating the height of the seat.

### Evaluation of the transparency of ALEx

As previously showed [21], the movements performed with the exoskeleton were more accurate but slightly less smooth. The higher accuracy could be ascribable to a deeper attention caused by the unusual situation of executing movements wearing an exoskeleton, which could also enforce a higher number of movement corrections. Indeed, a decreased smoothness was also observed in reaching trajectories performed by healthy subjects using ABLE [51], a robotic device with a design similar to ALEx.

Furthermore, the use of the exoskeleton resulted in modifications of the joint kinematics: the abduction-adduction and the rotation of the shoulder were reduced, while the flexion-extension of the shoulder and elbow augmented, in particular in some directions (i.e., North and East). Remarkably, an increased range for the elbow and the shoulder motion was reported also by using ABLE [51].

These modifications were reflected also in the muscle coordination. Indeed, the reaching task proposed in the experiment involved a significant modulation of the shoulder muscles for the gravity compensation and of the elbow flexors and extensors [21, 31]. However, the use of ALEx induced a redistribution of the contribution of the muscle groups for the execution of the reaching task, as reflected by an enhanced activation of the most cervical segments of the spinal maps and by a reduction of the activity in the most thoracic segments. Indeed, the contribution of the muscles involved in the control of the shoulder and of the elbow extension during free movements was substituted by a higher activation of the back muscles and of the elbow flexors. The altered muscle activity was reflected also in the absence of the DMED and DPOS synergy, which was present in free movements, and in the presence of two additional muscle synergies involving the BICS.

These evidences suggested a modification of the strategy adopted by the subjects while using the exoskeleton that could be due to postural adjustments or to the constrains provided by the structure of the exoskeleton. Indeed, despite in both conditions we asked the subjects not to move their back, when wearing ALEx, the subjects were seating in a chair ensuring the posture of the back with seat belts, while for the free movements the back was unconstrained. Another possible cause could be a not optimal compensation of the shoulder’s flexion-extension and rotation. Indeed, these DoFs are characterized by a shorter transmission and a consequent higher rigidity then the other joints. Moreover, a misalignment between the exoskeleton and the human limb, in particular at the level of the elbow joint, could cause the generation of undesirable interaction forces.

### Effects of the control strategies and of the EE trajectories on movement execution and muscular activity

Active and passive movements are primary control paradigms adopted for robotic therapy [19]. Elucidating the differences between active and passive training may help understanding and improving robot-assisted therapy, since the knowledge about the promotion of motor learning and recovery by active and passive exercises is still poor [52]. Therefore, in this work we investigated the effects of passive movements (i.e., during assisted control) on EE and joint kinematics and on muscle activity and coordination.

Our results confirmed our preliminary hypothesis, and they showed that passive arm movements induce similar effects of fully supported ones [31, 42]: in general, the passive training with ALEx elicited a significant muscle activity in most of the muscle groups, even though the activity was lower than during active reaching. However, some differences were present in the spatio-temporal organization of muscle synergies (in particular for S2 and S3), proving that despite the preservation of muscle coordination and a similar overall spinal activity, the assistive modality would achieve a muscle output with a different organization of the spinal circuitries with respect to the free movements and the passive modality. These differences seemed to be reduced when passive movements were elicited by trajectories previously recorded from the subjects. Therefore, desired trajectories for the passive training should be carefully evaluated in the robotic therapy. As a matter of fact, the execution of natural trajectories, which were more complex than the straight lines, promoted the activation of two additional muscle synergies. Indeed, it has been observed that a large number of muscle synergies occurred with a greater independence among the activation patterns of each muscle necessary to perform complex movements [8, 34].

Despite the differences between the control strategies, our findings suggest that all assistive modalities show similar control strategies markedly different from passive modalities.

How active and passive movements are processed and managed by the CNS is still an open question. Studies focusing on brain organization during passive and active movements showed that common brain networks are involved in the two conditions [53]. In particular, the brain activity is almost identical in location and intensity in the primary sensory-motor cortex during both passive and active elbow flexio-extension in healthy subjects [54]. However, what happens at the level of spinal circuitries has never been investigated so far. Our results suggest that afferent inputs resulting from passive movements (i.e., during assisted control) would be processed by different neural networks in the spinal cord than those specific for voluntary movements (passive modality). Indeed, in case of passive movements, the activity may occur in spinal circuitries involved in the process of afferent inputs and in the generation of reflexes, while, in case of voluntary movements, the activity of the circuitries that process the afferent inputs may occur in synergy with descending control pathways that modulate and integrate peripheral inputs [53].

In stroke patients both the descending control inputs and the sensory-motor feedback may be severely affected [55], resulting in different control strategies than those observed in healthy subjects, and this may be reflect by different muscle synergies and spinal maps related to the affected side.

Further investigations in an enlarged cohort of healthy and neurological subjects and by using other imaging techniques are necessary to confirm and deepen our preliminary findings.

### Considerations for the use of ALEx in rehabilitation

Our results on healthy subjects showed that ALEx in passive and assistive modality supports the upper limb reducing the abduction-adduction and rotation of the shoulder while increasing the flexion-extension of shoulder and elbow, which limited the activity of postural muscles and of the abductor of the shoulder and enhanced the activity of the elbow flexors. From these results, we expect that post-stroke subjects will be able to perform active movements inside ALEx. However, using the exoskeleton in passive modality the kinematics and the muscle activity of stroke subjects will significantly differ from those of healthy subjects. During actively assisted movements, instead, we expect that trajectories and muscle activity will be relatively similar between healthy subjects and patients, since the robot guides and assists the execution of the movements.

Clinically, robotic devices able to provide measured levels of gravity compensation on the upper limb proved to have a positive effect on the rehabilitative outcomes of post-stroke subjects, in particular of the most severe ones, because they improve the range of motion of the affected arm during the execution of vertical movements that involve shoulder elevation [56–58]. This improvement seems to be ascribable to a reduction of the abnormal coupling between shoulder abduction and elbow flexion [58–60]. The changes in the strategy adopted by the subjects when wearing ALEx reassembled the effects of a shoulder weight support and, thus, they could be considered positive for stroke rehabilitation. In addition, the possibility to act on muscle synergies controlling the shoulder seems to have a clinical validity, because they are the ones presenting higher alteration after stroke [28, 60, 61], and their modification usually occurs in concomitance with motor improvements [28].

In general, further investigations on post-stroke subjects would be necessary to confirm and test the preliminary findings of this work.

### Advantage of ALEx with respect to existing devices

Existing exoskeletons for upper limb rehabilitation often present consistent weight and dimensions (for instance 18.8 Kg for the ARMin III [62], 12 Kg for the MGA-Exoskeleton [63], and 6.8 Kg for the Rosen’s exoskeleton [64]) that augment the complexity of the control to compensate the high inertia, restrict the training workspace, provide unnatural sensory-feedbacks, and allow limited variety of exercises.

In the case of ALEx, the positions of the motors in the backpack strongly reduced the weight of the robotic arm (i.e., 4.5 Kg) and, thus, the inertia and friction of the moving parts, in particular at high speed. However, the transmission cable system reduces the possibility to adjust the length of the robotic arm, and this may cause a misalignment of the anatomical and robotic axis. The latter can limit the shoulder translation forcing the adoption of different strategies during reaching towards high targets, and it can be the cause of the excessive activation of the biceps muscles.

Albeit the human-machine contacts are minimal with respect to other devices [62] and they reproduce therapists’ behaviors [65] reducing the unnatural sensory feedback, some improvements may further optimize the transparency of the system. In particular, the shoulder translation could be facilitated with the integration of a passive joint for the shoulder elevation, and the adjustability of the robotic arm length could improve the alignment of the exoskeleton with the human arm, reducing the flexion/extension compensation at the shoulder and at the elbow.

### Study limitations

This study involves a small and relative young cohort of participants that reduces the strength of the statistical findings and their transferability to an elderly patient population. However, given the limited variability across subjects, we expect that the results would not significantly change considering a larger group. Moreover, to mimic the performances of elderly subjects we adopted a relatively slow speed. Indeed, dominant arm paths remained similar between young and elderly groups at low speed [66, 67].

The third session, which had the aim to show the possible active controls of ALEx, included a smaller cohort of subjects. However, the results did not reveal intra-subject differences between the two control modes revealing unnecessary a deeper analysis in this direction also because the findings related to these control strategies will be difficult to translate in a clinical environment with hemiplegic patients.

The differences in movement execution and muscle activity that we observed with and without exoskeleton cannot be merely ascribable to the use of the device and its transparency, but they may be also related to our specific set up and to the different instruments used to perform the measures in the two conditions. For instance, the presence of other elements in the workspace (like the initial position) and the complexity of the reaching task (more directions and planes) could contribute to the adoption of a particular kinematic and muscular strategy to perform the motor task when wearing the exoskeleton. However, the same task and setup were already adopted to investigate upper limb muscle synergies underlying reaching in different conditions [31, 44, 50] and are often used in post-stroke rehabilitation. Therefore, we proposed it to favor the comparison of our results with the existing literature and with clinical results.

## Conclusions

Our preliminary results on healthy subjects show the potentialities of ALEx used in different rehabilitative strategies for assisting the upper limb during reaching movements and for eliciting a muscle activity able to affect the spinal circuitries. The results show that during the execution of movements completely assisted by the exoskeleton, the fundamental muscle coordination was maintained, but the level of activity of the arm muscle groups was lower than in movements performed actively by the subjects. Moreover, the choice of the trajectories proposed in the upper limb motor tasks seems to have a significant impact on the elicited organization of both the muscles and the spinal circuitries.

## Abbreviations

ALEx: Arm Light Exoskeleton
BRA: brachialis
BRAD: brachioradialis
BICL: biceps brachii long head
BICS: biceps brachii short head
bpm: beats per minute
C2-T1: cervical and thoracic segments in the spinal cord
CoA: Center of Activity
DANT: anterior deltoid
d_COA_: mean distance between the center of activity of two spinal maps
d_EE_: point-to-point Euclidian distance
DMED: medial deltoid
DPOS: posterior deltoid
DoF: degree of freedom
DOT_SYN_: scalar product between the weighting coefficients of muscle synergies
d_joint_: the absolute distance between joint angular excursions
EE: end-effector
EL-Flx: elbow flexion
EMG: electromyographic
EPFL: École Polytechnique Fédérale de Lausanne
FO-Pro: forearm prono-supination
HMI: human machine interface
INFRA: infraspinatus
LAT: latissimus dorsi
MN: motoneuronal
MVC: maximum voluntary contraction
nMD: mean distance
NE: condition without the exoskeleton
NNMF: non-negative matrix factorization
nPK: number of peaks in the speed profile
PEC: pectoralis major
PERCRO: Perceptual Robotics laboratory
PRO: pronator teres
RHO: rhomboid major
R_Map-EMG_: 2D correlation coefficient between two spinal maps
R_Map-SYN_: 2D correlation coefficient between the spatio-temporal organization of muscle synergies
RMS_EMG_: root mean square of the EMG data
RMS_SYN_: root mean square of the timing activation of muscle synergies
R_joint_: Pearson’s correlation coefficient between two joint angular excursions
SENIAM: surface electromyography for non-invasive assessment of muscles
SH-Abd: shoulder abduction
SH-Flx: shoulder flexion
SH-Rot: shoulder rotation
TRILAT: lateral head of triceps brachii
TRILONG: long head of triceps brachii
TRAPM: medial trapezius
TRAPS: superior trapezius
VAF: variance accounted for
WEA: condition wearing the exoskeleton in the active modality, session 2
WEAE: condition wearing the exoskeleton in the active modality with the control at the end-effector, session 3
WEP1: condition wearing the exoskeleton in the passive modality, session 1
WEP2: condition wearing the exoskeleton in the passive modality, session 2
WEP3: condition wearing the exoskeleton in the passive modality, session 3
WEAJ: condition wearing the exoskeleton in the active modality with the control at the joints, session 3
WR-Flx: wrist flexion
